# A Prospective Cohort Study of Bioavailable 25-Hydroxyvitamin D Levels as a Marker of Vitamin D Status in Nontuberculous Mycobacterial Pulmonary Disease

**DOI:** 10.3390/nu13082524

**Published:** 2021-07-23

**Authors:** Byoung-Soo Kwon, Kyunghoon Lee, Eun-Sun Kim, Sun-Hee Jun, Sung-Yoon Lim, Myung-Jin Song, Yeon-Wook Kim, Yeon-Joo Lee, Jong-Sun Park, Young-Jae Cho, Ho-Il Yoon, Choon-Taek Lee, Junghan Song, Jae-Ho Lee

**Affiliations:** 1 Department of Internal Medicine, Division of Pulmonary and Critical Care Medicine, Seoul National University Bundang Hospital, 82 Gumi-ro 173 Beon-gil, Bundang-gu, Seongnam-si 13620, Gyeonggi-do, Korea; bskwon82@gmail.com (B.-S.K.); apolio3700@gmail.com (E.-S.K.); nucleon727@gmail.com (S.-Y.L.); mjsong8705@snubh.org (M.-J.S.); kimyw@snubh.org (Y.-W.K.); yjlee1117@snubh.org (Y.-J.L.); jspark.im@gmail.com (J.-S.P.); lungdrcho@snubh.org (Y.-J.C.); dextro70@gmail.com (H.-I.Y.); ctlee825@gmail.com (C.-T.L.); 2 Department of Laboratory Medicine, Seoul National University Bundang Hospital, Seongnam-si 13620, Gyeonggi-do, Korea; khlee59023@gmail.com (K.L.); sunnyeyo@snubh.org (S.-H.J.); songjhcp@snu.ac.kr (J.S.)

**Keywords:** nontuberculous mycobacterial, vitamin D, bioavailable

## Abstract

Research on vitamin D in patients with nontuberculous mycobacterial (NTM) pulmonary disease (PD) is limited. We aimed to compare the vitamin D parameters of patients with NTM-PD to those of a healthy control group, and to assess the possible predictive markers for a clinical response. We prospectively enrolled 53 patients with NTM-PD between January 2014 and December 2016. The clinical data and vitamin D indices, including total, free, bioavailable 25-(OH)D, and vitamin D binding protein (VDBP) genotyping, were measured at baseline and six months after enrollment. An external dataset of 226 healthy controls was compared with the NTM-PD group. The mean age of subjects was 53 years; 54.5% were male. The NTM-PD group was older, predominantly female, and had a lower body mass index (BMI) than the controls. The proportion of patients with vitamin D concentration <50 nmol/L was 52.8% in the NTM-PD group and 54.9% in the control group (*p* = 0.789). The bioavailable 25-(OH)D concentrations of the NTM-PD group and the controls were similar (6.9 nmol/L vs. 7.6 nmol/L, *p* = 0.280). In the multivariable analysis, bioavailable 25-(OH)D concentrations were associated with NTM-PD, adjusting for age, sex, BMI, and VDBP levels. Bioavailable 25-(OH)D concentrations were significantly associated with susceptibility to NTM-PD, but not with treatment outcomes. Lower bioavailable 25-(OH)D might be a risk factor for NTM-PD.

## 1. Introduction

Nontuberculous mycobacteria (NTM) are ubiquitous organisms that cause chronic pulmonary disease (PD) [1]. The global burden of NTM-PD is increasing, and the incidence of NTM-PD surpasses that of tuberculosis [2]. A population-based study showed that NTM-PD is associated with significant morbidity and mortality [3]. Although a multidrug regimen with prolonged duration is recommended for the treatment of NTM-PD, the treatment outcomes are still suboptimal [4]. Moreover, malnutrition, which is frequently seen in patients with NTM-PD [5,6,7,8], has been shown to be a poor prognostic factor by previous studies [9,10].

Vitamin D plays a role in innate immunity as well as in calcium homeostasis and bone metabolism [11,12,13,14]. The active form of vitamin D, 1,25-(OH)_2_D_3_, which is mainly hydroxylated in the kidney from an inactive form of 25-(OH)D, is also converted by a macrophage [15]. This activated vitamin D augments innate immunity by the fusion of phagolysosomes [11] and activates the intracellular signaling pathway by binding vitamin D binding protein (VDBP). Along with vitamin D, VDBP exerts immunological effects directly via neutrophil chemotaxis and a macrophage-activating factor (MAF), and indirectly by binding to activated vitamin D [12,16].

Previous studies have consistently demonstrated that vitamin D deficiency has been considered an associated factor for tuberculosis (TB) [17,18,19]. However, studies of vitamin D status in patients with NTM-PD have been scanty and showed inconclusive results [20,21]. Recently, there has been increasing evidence suggesting that bioavailable (albumin-bound plus free form) and free 25-(OH)D, not total 25-(OH)D, were more likely to be a predictive biomarker for vitamin D status [22,23]. We designed a comprehensive study to compare the total and bioavailable vitamin D of patients with NTM-PD to those of healthy controls. Additionally, we examined which parameters were related to clinical outcomes and used longitudinal data to assess whether those parameters changed before and after the treatment.

## 2. Materials and Methods

### 2.1. Participants

This was a single-center, observational, prospective study that consecutively enrolled patients with NTM-PD between January 2014 and December 2016 at a tertiary referral center in South Korea. Patients diagnosed with NTM based on the American Thoracic Society and Infectious Diseases Society of America guideline [1] were included regardless of the treatment status. At screening, patients with NTM-PD were assessed for eligibility and excluded if they were taking vitamin D supplements. Further exclusion criteria were: a total 25-(OH) D ≥ 125 nmol/L; withdrawal of consent; loss to follow-up; and violation of protocol.

To compare the vitamin D indices with healthy controls, we combined the external clinical dataset of healthy controls undergoing a health screening program in March 2019 [24].

Informed consent was obtained from all participants except for the healthy controls. In the case of the control group, informed consent was waived because the data was anonymized and retrospectively obtained pooled data. The Institutional Review Board of Seoul National University Bundang Hospital approved the study protocol (IRB No. B-1204-150-012).

### 2.2. Study Protocol

Demographic, laboratory, mycobacterial, and bioelectrical impedance data of the participants were collected at the enrollment. Blood samples were frozen at −70 ℃ in liquid nitrogen until the analysis was performed. Six months after enrollment, blood sampling was repeated among all participants.

In patients with NTM-PD, sputum studies were conducted at baseline and followed by routine clinical practice. Treatment success was defined as the maintenance of sputum culture conversion for 12 months or more [1].

### 2.3. Ultra Performance Liquid Chromatography (UPLC)-Mass Spectrometry (MS)/MS Analysis

Following derivatization, separate reactions of hexane extraction, and trypsin digestion, treated serum samples were analyzed using the ACQUITY ultra-performance liquid chromatography system (Waters, Maple Street, Milford, MA, USA) and Xevo TQ-S mass analyzer (Waters) to measure 25-(OH)D_3_, 25-(OH)D_2_, 24,25-(OH)_2_D_3_, VDBP and its isoforms, and albumin. The bioavailable 25-(OH)D concentrations were calculated by using total 25-(OH)D, VDBP, and albumin results; these calculations were based on a formula developed by Vermeulen et al. [25]. More details are described in the previous study [24]. All the samples underwent UPLC-MS/MS analysis from 2019 to 2020.

### 2.4. Statistical Analysis

Continuous variables were compared by Student’s *t*-test and the Mann–Whitney U test. Categorical variables were compared by the chi-squared test and Fisher’s exact tests. The differences in vitamin D and VDBP between the patients and the healthy population were compared. The differences between the baseline and the 6-month vitamin D levels of patients with NTM were then analyzed by a paired *t*-test. For the subgroup analysis, the NTM group was further subdivided into two groups according to the treatment status. A logistic regression analysis was used to identify associated factors for NTM-PD. Variables that were identified in univariable analysis as significant were used in a multivariate analysis by backward log-likelihood methods. In all analyses, a *p*-value of <0.05 was considered significant. The statistical analysis was performed with IBM SPSS version 24 (SPSS, Inc., Chicago IL, USA) and Prism version 5 (GraphPad, San Diego, CA, USA).

## 3. Results

### 3.1. Baseline Characteristics

**Figure 1:**
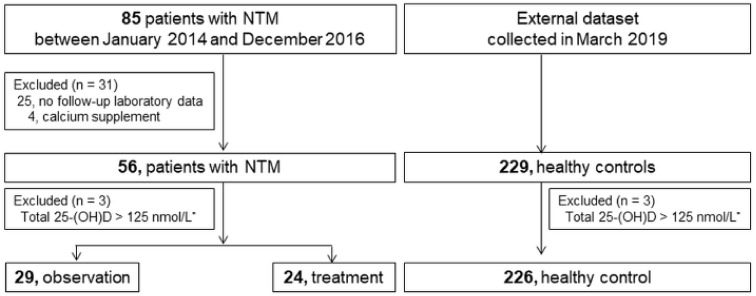
Flow-chart of included and excluded patients with nontuberculous mycobacterial pulmonary disease (NTM) and healthy controls. NTM, nontuberculous mycobacterium; * To minimize the possibility of taking dietary supplements, patients with a total 25-(OH)D ≥ 125 nmol/L were excluded.

**Figure 2:**
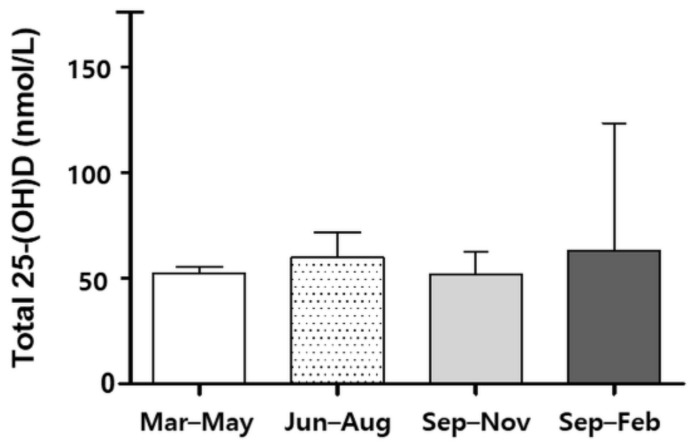
Total 25-(OH)D levels of 279 subjects between seasons.

A total of 226 healthy controls and 53 patients with NTM were included (Figure 1). The mean age of participants was 53 years, and 54.5% were male. Among the patients with NTM, 45.3% (24/53) had received anti-NTM treatment. Those in the NTM group were more likely to be older, female, and with a lower body mass index (BMI) compared with the healthy controls. *Mycobacterium avium* complex was the most common causative organism (90.6%), and nodular bronchiectatic form was the dominant pattern in the patients with NTM-PD (Table 1). The season of enrollment was significantly more likely to be spring, because all the data of the healthy subjects were collected in March. According to the sampling season, the total 25-(OH)D levels were similar among the total subjects (*p* = 0.523, Figure 2). No significant association was observed between the sampling season and the total 25-(OH)D level in patients with NTM-PD (*p* = 0.690, Figure S1).

### 3.2. Vitamin D and Vitamin D Binding Protein

At baseline, VDBP levels of patients with NTM-PD were significantly higher than those of the controls. The group-specific component (Gc) variants were not different between the groups. Gc 1f/Gc2 was the most common, accounting for 29.7% of the total. The Gc1f genotype was the most frequently seen in both subject groups (Table 2).

**Figure 3:**
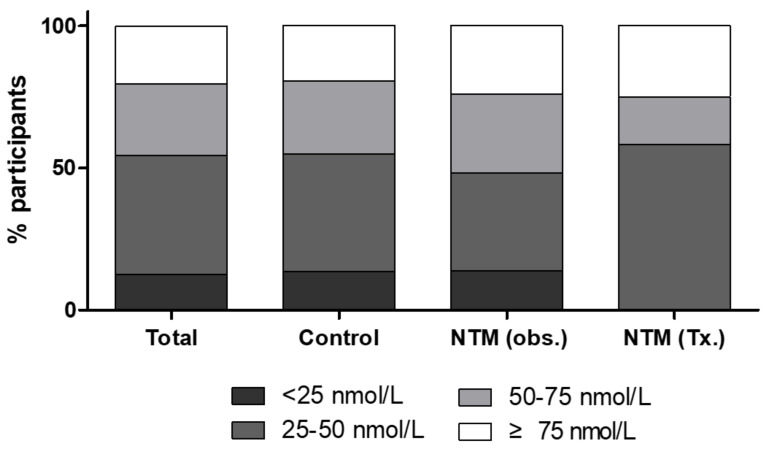
Distribution of vitamin D concentrations of patients with nontuberculous mycobacterial (NTM) pulmonary disease and healthy controls; NTM, nontuberculous mycobacterium; obs, observation group; Tx, treatment group.

**Figure 4:**
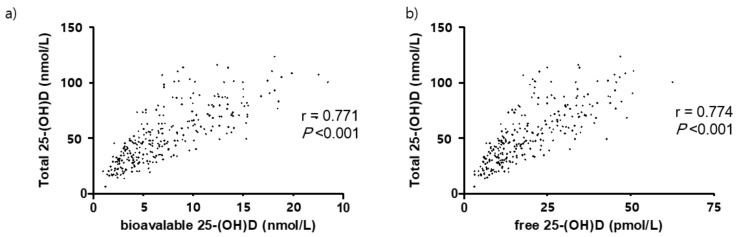
Correlation between total, bioavailable, and free 25(OH)D in 279 participants. (**a**) Bioavailable 25-(OH)D, (**b**) free 25-(OH)D.

**Figure 5:**
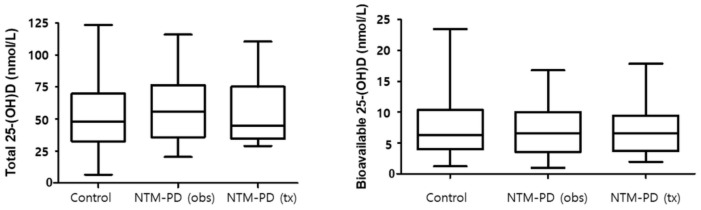
Total and genotype-specific bioavailable 25-(OH)D of patients with nontuberculous mycobacterial (NTM) pulmonary disease and healthy controls. NTM-PD, nontuberculous mycobacterial pulmonary disease; obs, observation group; tx, treatment group; Box plot shows median (central line), 25% and 75% quartile ranges.

Approximately 54.4% of participants were vitamin D deficient, with deficiency defined as having a total 25-(OH)D less than 50 nmol/L (Figure 3). Significant correlations between the total 25-(OH)D and the free and bioavailable forms were observed (Figure 4). At baseline, total 25-(OH)D and free and bioavailable vitamin D levels showed no significant differences between the NTM and the control group (Table 3, Figure 5). The differences between the baseline and the follow-up data showed no significant change in those NTM-PD patients in the group under observation. However, in the treatment group of patients with NTM-PD, the total 25-(OH)D levels were significantly decreased after 6 months of treatment. These changes were not observed in the free or bioavailable 25-(OH)D concentrations (Table 3).

### 3.3. Association Factors and Clinical Outcome of NTM-PD

In the multivariable analysis, bioavailable 25-(OH)D was identified as a risk factor for NTM-PD after adjusting for age, sex, BMI, and VDBP levels (Table 4). When propensity score matching was used at a ratio of 1:1 for sex, age, and BMI (case = 45 vs. control = 45), bioavailable 25-(OH)D levels still remained a significant factor associated with susceptibility to NTM-PD (Tables S1–S4).

Among the 24 patients with NTM who received treatment, 14 (58.3%) had treatment success. Demographic factors, acid-fast bacilli (AFB) smear positivity, NTM species, and radiologic types were not different between the two groups (Table 5).

## 4. Discussion

In this study, we measured VDBP and vitamin D levels in patients with NTM-PD and compared them with those of healthy controls. The most important point of our study was that serum bioavailable and free vitamin D levels were measured for the first time in patients with NTM-PD. We found that although the free and bioavailable 25-(OH)D levels of patients with NTM-PD were similar to those of healthy controls, bioavailable vitamin D was associated with susceptibility to NTM when adjusting for age, sex, BMI, and VDBP concentrations. During the follow-up, bioavailable 25-(OH)D levels were not significantly changed even after the treatment. We also found that VDBP concentrations were higher in the NTM group, even though Gc variants were similar between the patients with NTM and the healthy controls.

In the current study, we measured total 25-(OH)D using LC/MS-MS methods and found that the concentrations were similar between the patients with NTM and the healthy controls. It has been known that the vitamin D level is significantly negatively related with BMI and age [26,27]. Nevertheless, the results of our non-matching and matching analysis showed that vitamin D concentrations were not different between the patients with NTM-PD and the healthy controls. Previous studies of the association between vitamin D level and NTM-PD have shown inconsistent results. Jeon et al. reported that vitamin D levels were significantly lower in patients with NTM-PD even after adjusting confounding factors (10.7 vs. 13.7 ng/mL) [20]. In their study, the concentration of vitamin D was measured using an enzyme-linked immunosorbent assay (ELISA) kit. In contrast, Oh et al. reported that vitamin D concentrations of NTM-PD measured by LC-MS/MS methods were not significantly different compared to those of the controls (20.2 vs. 20.3 ng/mL, *p* = 0.678) [21]. These inconsistencies might be explained by the differences in measuring methods. It has been reported that the 25-(OH)D concentrations were higher when measured by LC/MS methods than when measured by ELISA [24]. Thus, our results were supported by the study of Oh et al. However, far more importantly, we also found that bioavailable 25-(OH)D levels were independently associated with NTM-PD after adjusting for covariates.

Approximately 85% of 25-(OH)D binds to VDBP, and the remaining 15% binds to albumin. The bioavailable (binds to albumin plus free form) or free 25-(OH)D only has a small portion of the total 25-(OH)D [28]. Theoretically, these free or bioavailable vitamins are linked to exert biological activity. Recent studies suggest that free or bioavailable 25-(OH)D, rather than total 25-(OH)D, is a more relevant parameter for predicting vitamin D status [22,29]. In our study, a lower concentration of bioavailable 25-(OH)D was significantly associated with susceptibility to NTM-PD. Moreover, bioavailable 25-(OH)D showed relatively stable changes compared with the total 25-(OH)D levels. Taken together, our findings suggest that bioavailable 25-(OH)D is a potential marker of vitamin D status in patients with NTM-PD. However, the subgroup analysis showed that bioavailable 25-(OH)D was similar between the treatment success and failure groups. Further studies with a large sample size are required to confirm the feasibility of bioavailable 25-(OH)D as a predictive marker for the treatment of NTM-PD.

In our study, VDBP concentration was significantly higher in the NTM group than in the control group, a finding consistent with previous research. For instance, Kim et al. showed that VDBP levels in patients with NTM-PD were higher than those in healthy controls [30]. It is known that VDBP has a functional role in neutrophil chemotaxis and macrophage activation [16]. Despite limited evidence in NTM-PD, several studies have demonstrated that VDBP concentrations were elevated in other lung diseases, such as chronic obstructive lung disease or pulmonary tuberculosis [31,32]. Given that the upregulation of VDBP binding sites and the enhancement of VDBP transcription are activated by neutrophil or pro-inflammatory cytokines [33], increased VDBP might be related to lung inflammation.

Regarding the Gc genotype, our results showed no difference in the frequency between the NTM-PD group and the healthy controls. In inflammatory responses, Gc proteins are hydrolyzed by B cells and T cells, and produce Gc-derived MAF. Gc-derived MAF has clinical usefulness regarding macrophage and phagocyte activation [34]. According to the previous study, Gc2 has less ability to be converted into MAF than Gc1 [35]. Thus, a reasonable hypothesis is that patients with Gc2 are more likely to be susceptible to certain infections. A study by Martineau et al. showed inconsistent results of the association between the Gc genotype and susceptibility to TB infection according to geographic region [36]. In addition, Ko et al. reported that Gc genotype frequency differed between race/ethnic groups [24]. Thus, multifactorial causes, including geographic region and ethnic differences, should be considered to evaluate the association between the VDBP polymorphism and specific disease entities, such as NTM-PD.

There were some limitations to our study. First, the study was conducted at a single center with a small number of participants. However, the demographics and vitamin D levels of patients with NTM-PD were similar to those in previous studies [21]. Second, despite the prospective design of the current study, information on the administration of vitamin D supplements was not fully scrutinized. Although four patients were excluded from the analysis due to a prescription history of vitamin D supplements, there was the possibility that patients who were taking dietary supplements were not excluded, particularly if patients who did not know whether they were taking supplements were enrolled. To minimize this possibility, we used the criterion of excluding patients with a total 25-(OH)D ≥125 nmol/L. However, this value was arbitrarily defined. Thus, the results should be interpreted with caution. Finally, other factors affecting vitamin D levels, such as sunlight exposure, dietary habit, and seasonal variation, were not evaluated.

## 5. Conclusions

We observed that bioavailable 25-(OH)D was an independent risk factor for NTM-PD after adjusting for covariates. Our results suggest that bioavailable 25-(OH)D might be a possible biomarker of the vitamin D status of patients with NTM-PD. In addition, although bioavailable 25-(OH)D did not differ significantly between the treatment success and failure groups, further studies on the prognostic role of bioavailable 25-(OH)D in the treatment of NTM-PD are required.

## Figures and Tables

**Table 1:** Comparison of baseline characteristics of participants according to the diagnosis.

	Total (*n* = 279)	Control (*n* = 226)	NTM (*n* = 53)	*p*-Value
Age	53.0 ± 11.7	51.0 ± 11.1	65.6 ± 10.5	<0.001
Sex, male	152 (54.5)	136 (60.2)	16 (30.2)	<0.001
BMI (kg/m^2^)	23.6 ± 3.1	24.1 ± 3.0	21.5 ± 2.6	<0.001
Causative organism				
* M. avium* complex			48 (90.6)	
* M. abscessus* complex			4 (7.5)	
* M. kansasii*			1 (1.9)	
Radiologic type				
Fibrocavitary	10 (18.9)		10 (18.9)	
Nodular bronchiectatic	43 (81.1)		43 (81.1)	
Positive AFB smear	17 (32.1)		17 (32.1)	
Sampling season				<0.001
Spring (March–May)	233 (83.5)	226 (100)	7 (13.2)	
Summer (June–August)	21 (7.5)	0 (0.0)	21 (39.6)	
Autumn (September–November)	21 (7.5)	0 (0.0)	21 (39.6)	
Winter (December–February)	4 (1.4)	0 (0.0)	4 (7.5)	

Data are expressed as mean ± standard deviation or number (%); NTM, nontuberculous mycobacterium; BMI, body mass index; *M. avium*, *Mycobacterium avium*; *M. abscessus*, *Mycobacterium abscessus*; *M. kansasii*, *Mycobacterium kansasii*; AFB, acid-fast bacilli.

**Table 2:** Allele frequency of VDBP isoforms and VDBP concentrations in study subjects.

	Total (*n* = 279)	Control (*n* = 226)	NTM (*n* = 53)	*p*-Value
Diplotypes				0.818
Gc1f/Gc2	83 (29.7)	67 (29.6)	16 (30.2)	
Gc1s/Gc1f	60 (21.5)	48 (21.2)	12 (22.6)	
Gc1f/Gc1f	59 (21.1)	47 (20.8)	12 (22.6)	
Gc1s/Gc2	40 (14.3)	36 (15.9)	4 (7.5)	
Gc2/Gc2	22 (7.9)	18 (8.0)	4 (7.5)	
Gc1s/Gc1s	15 (5.4)	10 (4.4)	5 (9.4)	
Haplotypes				0.441
Gc1f	261 (46.8)	209 (46.2)	52 (49.1)	
Gc1s	130 (23.3)	104 (23.0)	26 (24.5)	
Gc2	167 (29.9)	139 (30.8)	28 (26.4)	
VDBP (μg/mL)	224.0 ± 41.9	221.0 ± 31.0	235.5 ± 70.1	0.022

Data are expressed as mean ± standard deviation or number (%); VDBP, vitamin D binding protein; NTM, nontuberculous mycobacterium; Gc, group-specific component.

**Table 3:** Baseline and 6-month follow-up of total, bioavailable, and free 25-(OH)D levels in study subjects.

	Control (*n* = 226)	NTM (*n* = 53) *	NTM (*n* = 53)			
			Observation (*n* = 29)		Treatment (*n* = 24)	
			Baseline	6-Month	Baseline	6-Month ^†^
Total 25-(OH)D (nmol/L)	52.2 ± 24.9	55.9 ± 25.7	56.3 ± 26.1	57.4 ± 26.8	55.5 ± 25.7	48.2 ± 25.5 ^†^
25-(OH)D_2_	1.0 ± 0.8	1.5 ± 2.0	1.6 ± 2.5	1.6 ± 1.8	1.3 ± 0.8	1.1 ± 0.9 ^†^
25-(OH)D_3_	51.2 ± 24.9	54.5 ± 25.7	54.7 ± 26.1	55.8 ± 27.2	54.2 ± 25.8	47.1 ± 25.7 ^†^
Genotype-specific						
Free 25-(OH)D (pmol/L)	19.5 ± 11.9	19.5 ± 10.9	19.3 ± 10.9	19.0 ± 10.9	19.8 ± 11.1	17.3 ± 10.3
Bioavailable 25-(OH)D (nmol/L)	7.6 ± 4.7	6.9 ± 3.9	6.9 ± 4.0	6.6 ± 3.5	6.8 ± 4.0	6.0 ± 3.5
24,25-(OH)_2_D_3_ (nmol/L)	4.4 ± 2.9	3.9 ± 3.0	4.1 ± 3.0	3.8 ± 2.7	3.7 ± 3.0	2.3 ± 1.9 ^†^

Data are expressed as mean ± standard deviation; NTM, nontuberculous mycobacterium; * No statistically significant differences are observed between the NTM-PD and healthy control groups; † *p*-value < 0.05, between baseline and follow-up after 6 months in patients with NTM-PD.

**Table 4:** Risk factors associated with NTM-PD among study subjects.

	Univariable Analysis		Multivariable Analysis	
	OR (95% CI)	*p*-Value	OR (95% CI)	*p*-Value
Age	1.100 (1.063–1.139)	<0.001	1.134 (1.088–1.183)	<0.001
Sex, male	0.286 (0.150–0.545)	<0.001	0.357(0.161–0.794)	0.012
BMI	0.707 (0.619–0.807)	<0.001	0.677 (0.578–0.793)	<0.001
Total 25-(OH)D *	1.015 (0.985–1.045)	0.331		
Genotype-specific				
Free 25-(OH)D	1.001 (0.939–1.067)	0.980		
Bioavailable 25-(OH)D	0.908 (0.763–1.081)	0.280	0.736 (0.588–0.921)	0.007
VDBP	1.008 (1.000–1.015)	0.062	–	–
24,25-(OH)_2_D_3_	0.864 (0.657–1.137)	0.297	–	–

NTM, nontuberculous mycobacterium; PD, pulmonary disease; OR, odds ratio; CI, confidential interval; BMI, body mass index; VDBP, vitamin D binding protein; * Total 25-(OH)D was excluded in the multivariable analysis due to the close correlation with bioavailable 25-(OH)D (r = 0.771, *p* < 0.001).

**Table 5:** Vitamin D parameters in patients with NTM-PD according to the treatment outcomes.

	Treatment Outcomes		*p*-Value
	Success (*n* = 14)	Failure (*n* = 10)	
Age	61.0 (55.5–66.0)	60.5 (49.5–65.3)	0.703
Sex, male	4 (28.6)	3 (30.0)	>0.999
BMI	21.4 (20.2–22.3)	21.4 (18.8–23.6)	0.884
AFB smear positivity	5 (35.7)	7 (70.0)	0.214
Causative organism			0.525
* M. avium* complex	13 (90.9)	10 (100.0)	
* M. Kansasii*	1 (9.1)	0 (0.0)	
Radiologic types			>0.999
Fibrocavitary	5 (35.7)	4 (40.0)	
Nodular bronchiectatic	9 (64.3)	6 (60.0)	
Total 25-(OH)D	64.0 (40.1–82.4)	39.5 (32.2–47.7)	0.143
25-(OH)D_2_	1.0 (0.8–1.6)	1.1 (0.9–1.3)	0.429
25-(OH)D_3_	62.6 (38.5–81.6)	38.4 (30.0–46.9)	0.143
Genotype-specific			
Free 25-(OH)D	21.1 (12.9–32.3)	16.7 (7.4–23.6)	0.242
Bioavailable 25-(OH)D	7.0 (4.3–10.7)	6.1 (2.4–7.2)	0.242
24,25-(OH)_2_D_3_	3.1 (1.7–7.7)	2.2 (1.4–4.1)	0.192

Data are expressed as median (interquartile range); NTM, nontuberculous mycobacterium; PD, pulmonary disease; *M. avium*, *Mycobacterium avium*; *M. abscessus*, *Mycobacterium abscessus*; *M. kansasii*, *Mycobacterium kansasii*; AFB, acid-fast bacilli.

## Data Availability

The data presented in this study are available on request from the corresponding author.

## Supplementary Materials

The following are available online at https://www.mdpi.com/article/10.3390/nu13082524/s1, Figure S1: Total 25-(OH)D levels in patients with NTM-PD according to the sampling season; Table S1: Comparison of baseline characteristics of matched cohort according to the diagnosis; Table S2: Baseline and 6-months follow-up of vitamin D indices in the matched cohort; Table S3: Risk factors associated with NTM-PD; Table S4: Vitamin D parameters in patients with NTM-PD according to the treatment outcomes.
