# Supplementary material for: A Prospective Cohort Study of Bioavailable 25-Hydroxyvitamin D Levels as a Marker of Vitamin D Status in Nontuberculous Mycobacterial Pulmonary Disease
Source: Nutrients. 2021 Jul 23;13(8):2524. doi: 10.3390/nu13082524 (PMC8400277; doi:10.3390/nu13082524)
Supplement: Supplementary file 1 [file nutrients-13-02524-s001.zip › nutrients-1303374-supplementary.pdf]

Supplementary Material

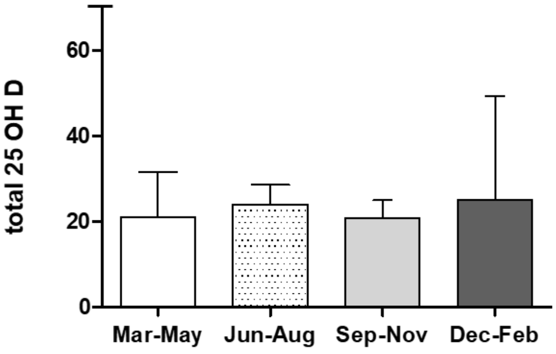

Figure S1. Total 25-(OH)D levels in patients with NTM-PD according to the sampling season

Table S1. Comparison of baseline characteristics of matched cohort according to the diagnosis.

	Total (n = 90)	Control (n = 45)	NTM (n = 45)	*P*-value
Age	60.4 ± 9.8	60.1 ± 9.7	60.8 ± 10.0	0.689
Sex, male	31 (3434)	16 (35.6)	15 (33.3)	<0.001
BMI (kg/m^2)	21.7 ± 2.2	21.6 ± 1.9	21.8 ± 2.5	0.778

Data are expressed as mean ± standard deviation or number (%). NTM, nontuberculous mycobacterium; BMI, body mass index;.

Table S2. Baseline and 6-months follow-up of vitamin D indices in the matched cohort

	Control (n=45)	NTM (n=45)	NTM (n = 45)			
			Observation (n=27)		Treatment (n=18)	
			Baseline	6-month	Baseline	6-month†
Total 25-(OH)D (nmol/L)	62.2 ± 26.8	57.3 ± 25.2	57.2 ± 26.2	58.3 ± 26.7	57.5 ± 24.4	48.1 ± 26.9†
25-(OH)D2	0.9 ± 0.6	1.4 ± 2.1	1.6 ± 2.6	1.5 ± 1.9	1.1 ± 0.5	0.9 ± 0.4
25-(OH)D3	61.3 ± 26.8	55.9 ± 25.3	55.6 ± 26.2	56.7 ± 27.1	56.4 ± 24.5	47.2 ± 27.0†
Genotype specific						
Free 25-(OH)D	22.9 ± 12.6	19.6 ± 10.2	19.3 ± 11.0	19.2 ± 10.7	19.9 ± 9.2	16.3 ± 10.4†
Bioavailable 25-(OH)D (nmol/L)	8.8 ± 5.0	6.9 ± 3.7*	6.9 ± 4.0	6.6 ± 3.5	6.9 ± 3.3	5.6 ± 3.4†
24,25-(OH)2D3 (nmol/L)	5.7 ± 3.5	4.1 ± 2.9*	4.2 ± 3.1	3.9 ± 2.7	3.9 ± 2.9	2.2 ± 2.0†

Data are expressed as mean ± standard deviation. NTM, nontuberculous mycobacterium * *P*-value < 0.05, between the NTM-PD and healthy control groups. † *P*-value < 0.05, between baseline and follow-up after 6 months in patients with NTM-PD

Table S3. Risk factors associated with NTM-PD

	Univariable analysis		Multivariable analysis	
	OR (95% CI)	*P*-value	OR (95% CI)	*P*-value
Age	1.007 (0.965–1.051)	0.738	–	–
Sex, male	0.906 (0.380–2.163)	0.824	–	–
BMI	1.028 (0.852–1.240)	0.775	–	–
Total 25-(OH)D	0.982 (0.943–1.022)	0.374		
Genotype specific				
Free 25-(OH)D	0.938 (0.856–1.029)	0.175		
Bioavailable 25-(OH)D	0.781 (0.611–0.999)	0.049	0.781 (0.611–0.999)	0.049
VDBP	1.012 (0.998–1.026)	0.096	–	–

NTM, nontuberculous mycobacterium; PD, pulmonary disease; OR, odds ratio; CI, confidential interval; BMI, body mass index; VDBP, vitamin D binding protein

Table S4. Vitamin D parameters in patients with NTM-PD according to the treatment outcomes

	**Treatment outcomes**		**P-value**
	**Success (n=13)**	**Failure (n=5)**	
Age	61.0 (54.0 – 65.0)	50.0 (46.0 – 62.0)	0.289
Sex, male	4 (30.8)	2 (40.0)	>0.999
BMI	21.1 (19.9 – 22.1)	22.5 (20.0 – 23.9)	0.289
AFB smear positivity	5 (38.5)	5 (100.0)	0.036
Causative organism			-
M. avium complex	12 (92.3)	5 (100.0)	
M. Kansasii	1 (7.7)	0 (0.0)	
Radiologic types			0.118
Fibrocavitary	4 (30.8)	4 (80.0)	
Nodular bronchiectatic	9 (69.2)	1 (20.0)	
Total 25-(OH)D	21.9 (16.0 – 31.2)	18.0 (13.5 – 31.0)	0.703
25-(OH)D_2	0.4 (0.3 – 0.5)	0.5 (0.4 – 0.5)	0.336
25-(OH)D_3	21.0 (15.3 – 30.9)	17.6 (13.1 – 30.5)	0.775
Genotype specific			
Free 25-(OH)D	8.3 (5.0 – 12.0)	7.0 (4.5 – 9.9)	0.924
Bioavailable 25-(OH)D	2.6 (1.7 – 4.0)	2.8 (1.6 – 3.4)	0.924
24,25-(OH)2D3	2.9 (1.6 – 7.0)	4.0 (1.6 – 4.4)	0.849

Data are expressed as median (interquartile range). NTM, nontuberculous mycobacterium; PD, pulmonary disease; *M. avium*, *Mycobacterium avium*; *M. abscessus*, *Mycobacterium abscessus*; *M. kansasii*, *Mycobacterium kansasii*; AFB, acid-fast bacilli
